# α-Synuclein Dimers Impair Vesicle Fission during Clathrin-Mediated Synaptic Vesicle Recycling

**DOI:** 10.3389/fncel.2017.00388

**Published:** 2017-12-11

**Authors:** Audrey T. Medeiros, Lindsey G. Soll, Isabella Tessari, Luigi Bubacco, Jennifer R. Morgan

**Affiliations:** ^1^The Eugene Bell Center for Regenerative Biology and Tissue Engineering, Marine Biological Laboratory, Woods Hole, MA, United States; ^2^Department of Biology, University of Padova, Padova, Italy

**Keywords:** dynamin, dynasore, endocytosis, lamprey, reticulospinal synapse

## Abstract

α-Synuclein is a presynaptic protein that regulates synaptic vesicle (SV) trafficking. In Parkinson’s disease (PD) and several other neurodegenerative disorders, aberrant oligomerization and aggregation of α-synuclein lead to synaptic dysfunction and neurotoxicity. Despite evidence that α-synuclein oligomers are generated within neurons under physiological conditions, and that altering the balance of monomers and oligomers contributes to disease pathogenesis, how each molecular species of α-synuclein impacts SV trafficking is currently unknown. To address this, we have taken advantage of lamprey giant reticulospinal (RS) synapses, which are accessible to acute perturbations via axonal microinjection of recombinant proteins. We previously reported that acute introduction of monomeric α-synuclein inhibited SV recycling, including effects on the clathrin pathway. Here, we report the effects of α-synuclein dimers at synapses. Similar to monomeric α-synuclein, both recombinant α-synuclein dimers that were evaluated bound to small liposomes containing anionic lipids *in vitro*, but with reduced efficacy. When introduced to synapses, the α-synuclein dimers also induced SV recycling defects, which included a build up of clathrin-coated pits (CCPs) with constricted necks that were still attached to the plasma membrane, a phenotype indicative of a vesicle fission defect. Interestingly, both α-synuclein dimers induced longer necks on CCPs as well as complex, branching membrane tubules, which were distinct from the CCPs induced by a dynamin inhibitor, Dynasore. In contrast, monomeric α-synuclein induced a buildup of free clathrin-coated vesicles (CCVs), indicating an inhibition of clathrin-mediated endocytosis at a later stage during the clathrin uncoating process. Taken together, these data further support the conclusion that excess α-synuclein impairs SV recycling. The data additionally reveal that monomeric and dimeric α-synuclein produce distinct effects on clathrin-mediated endocytosis, predicting different molecular mechanisms. Understanding what these mechanisms are could help to further elucidate the normal functions of this protein, as well as the mechanisms underlying PD pathologies.

## Introduction

α-Synuclein is a small (140 amino acid) presynaptic protein that interacts with synaptic vesicles (SVs) and regulates SV trafficking (Maroteaux et al., 1988; Murphy et al., 2000; Greten-Harrison et al., 2010; Vargas et al., 2014; Logan et al., 2017). Although its physiological function is still under investigation, overexpression or mutation of the α-synuclein gene, which lead to misfolding and aggregation of the protein, are linked to familial Parkinson’s disease (PD; Krüger et al., 1998; Spillantini et al., 1998; Lee and Trojanowski, 2006; Bendor et al., 2013; Dettmer et al., 2016; Lautenschläger et al., 2017). α-Synuclein overexpression and aggregation are also commonly observed in other neurodegenerative disorders, including Lewy body dementia (LBD), multiple systems atrophy and some variants of Alzheimer’s disease (Schulz-Schaeffer, 2010; Moussaud et al., 2014; Ingelsson, 2016). In animal models, overexpression of α-synuclein induces aberrant aggregation of the protein throughout neurons, causing numerous cellular defects including synaptic and mitochondrial impairment, which ultimately lead to neurodegeneration (Surguchov, 2008; Dawson et al., 2010; Scott et al., 2010; Nakamura et al., 2011; Nakamura, 2013; Lautenschläger et al., 2017).

In addition to somatic aggregation and Lewy body formation, pathophysiological accumulation of α-synuclein occurs in presynaptic nerve terminals, leading to synaptic defects (Schulz-Schaeffer, 2010; Scott et al., 2010; Unni et al., 2010; Spinelli et al., 2014). In LBD, the vast majority of aggregated α-synuclein localizes to synapses throughout the frontal cortex, while only a small fraction of aggregated α-synuclein is in the somatic Lewy bodies (Kramer and Schulz-Schaeffer, 2007; Schulz-Schaeffer, 2010). This synaptic aggregation correlates with greater cognitive deficits (Poletti et al., 2011; Irwin et al., 2013). In cultured mammalian neurons, overexpression of α-synuclein or exogenous application leads to measurable oligomerization and aggregation at presynapses (Scott et al., 2010; Volpicelli-Daley et al., 2011; Boassa et al., 2013; Spinelli et al., 2014). Similarly, a PD mouse model also exhibited significant synaptic aggregation of α-synuclein (Spinelli et al., 2014). α-Synuclein overexpression depletes synapses of several major presynaptic proteins, including synapsin (Nemani et al., 2010; Scott et al., 2010; Spinelli et al., 2014), an effect that has also been observed *post mortem* in LBD brains (Scott et al., 2010). A growing body of evidence indicates that excess α-synuclein, delivered acutely or by overexpression, inhibits neurotransmission primarily by impairing SV recycling and/or reclustering after endocytosis (Nemani et al., 2010; Scott et al., 2010; Busch et al., 2014; Xu et al., 2016; Eguchi et al., 2017), though effects on fusion pore kinetics during exocytosis were also recently reported (Logan et al., 2017). While it is clear that increased levels of α-synuclein impair SV trafficking, the underlying mechanisms remain unknown, though excess microtubule polymerization may play a role (Eguchi et al., 2017).

The classic model for α-synuclein toxicity postulates that aberrant folding of monomeric α-synuclein, or its overexpression, leads to formation of small toxic oligomers, aggregates and fibrils, resulting in deleterious effects throughout the neuron (Lee and Trojanowski, 2006). However, there is an ongoing debate about whether the native form of α-synuclein is monomeric or tetrameric, which brings this linear model into question (Bartels et al., 2011; Wang et al., 2011; Burré et al., 2013, 2015; Dettmer et al., 2015b). While it is generally agreed that neurons generate some α-synuclein oligomers under physiological conditions (Wang et al., 2014), and that altering the balance of monomers and oligomers contributes to disease pathogenesis, how each molecular species of α-synuclein (e.g., monomer, dimer, tetramer, etc.) impacts vesicle trafficking is unknown because oligomerization status is difficult to control in most overexpression models. In contrast, acute perturbations at large vertebrate synapses offer an amenable approach to this problem. Lamprey reticulospinal (RS) synapses are large, glutamatergic synapses that reside within the giant axons of the lamprey spinal cord, and they have been instrumental in elucidating the molecular mechanisms of SV trafficking (Pieribone et al., 1995; Shupliakov et al., 1997; Gad et al., 2000; Bloom et al., 2003; Brodin and Shupliakov, 2006). Using this preparation, we previously demonstrated that acute introduction of monomeric α-synuclein directly to presynapses severely inhibited SV endocytosis (Busch et al., 2014). The results were consistent with effects on both clathrin-mediated endocytosis, as well as bulk endocytosis (Busch et al., 2014). Similarly, acute dialysis of monomeric α-synuclein to the mammalian calyx of Held impaired vesicle endocytosis, whereas exocytosis was relatively unaffected (Xu et al., 2016; Eguchi et al., 2017). However, whether increasing other molecular species of α-synuclein affects SV trafficking, and how, is completely unknown.

The goal of this study was to determine how α-synuclein dimers affect SV trafficking. Do α-synuclein dimers produce any effects on SV trafficking? If so, do these effects parallel those caused by monomeric α-synuclein, or are they unique and distinct? Alternatively, are synapses able to tolerate high levels of α-synuclein dimers, unlike monomeric α-synuclein? Our experiments utilized two recombinant α-synuclein dimers (CC and NC dimers), which were previously reported to exhibit similar folding, aggregation and fibrillation properties to monomeric α-synuclein (Pivato et al., 2012). α-Synuclein CC dimer comprises two α-synuclein molecules covalently linked at their C-termini, while NC dimer brings together the N-terminus of one α-synuclein with the C-terminus of another, thus likely producing parallel and anti-parallel dimers that were implicated as physiologic forms in a recent study on endogenous α-synuclein multimers (Wang et al., 2014). Here, using lamprey synapses, we demonstrate that both α-synuclein dimers inhibited SV recycling from the plasma membrane, similar to previous observations with monomeric α-synuclein (Busch et al., 2014). However, unlike monomeric α-synuclein, which inhibits the uncoating of CCVs, the α-synuclein dimers selectively inhibited clathrin-mediated SV recycling at an earlier stage during the vesicle fission step. These effects were also distinct from those produced by an inhibitor of dynamin, the GTPase that drives vesicle scission from the PM. Taken together, these data further corroborate the finding that excess α-synuclein impairs SV endocytosis and illustrate a novel concept that α-synuclein monomers and dimers affect different steps in the endocytic pathway.

## Materials and Methods

### Recombinant Proteins and SDS-PAGE

**Figure 1:**
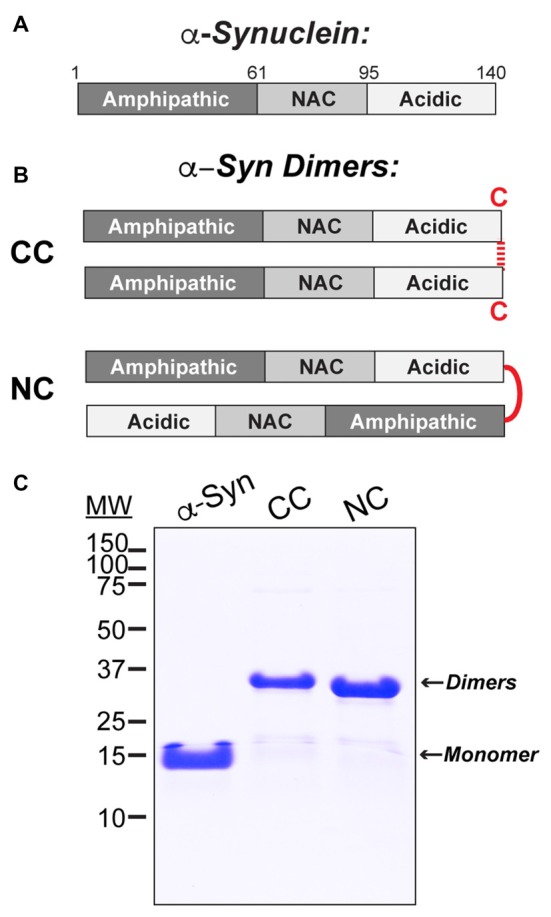
Stable recombinant α-synuclein dimers. **(A)** Domain diagram of monomeric human α-synuclein. **(B)** Diagrams of the α-synuclein dimers used in this study. CC dimer brings together the C-termini of two α-synuclein molecules via disulfide bonds. NC dimer was generated by engineering two copies of α-synuclein in tandem to generate a single polypeptide. **(C)** Coomassie stained 12% SDS-PAGE gel showing α-synuclein monomer and dimers and their respective molecular weights.

Wild type human α-synuclein (a.a. 1–140) was bacterially expressed in *E. coli* BL21 (DE3) from a pET28b plasmid (Novagen), as previously described (Figure 1A; Pivato et al., 2012). CC dimer was produced by adding terminal GC residues after a.a. 140 of the α-synuclein sequence, which allowed the molecules to be covalently linked by disulfide bonds at their C-termini (Figure 1B). NC dimer was produced by including two full-length copies of α-synuclein in the same plasmid, thus generating a dimer as a single polypeptide (Figure 1B). After expression and purification, α-synuclein dimers were isolated from monomers using anionic exchange and then lyophilized for long-term storage. Prior to use, the lyophilized dimers were resuspended in lamprey internal solution (180 mM KCl, 10 mM HEPES K^+^, pH 7.4). Any unwanted higher molecular weight α-synuclein oligomers or aggregates that may have formed during the lyophilization process were removed by passing the protein sample through a Microcon^®^ YM-100 centrifugal filter. Prior to injection, all preparations of monomeric and dimeric α-synuclein were run on a 12% SDS-PAGE gel and Coommassie stained in order to confirm purity and molecular weights (Figure 1C).

### Liposome Binding and Western Blotting

**Figure 2:**
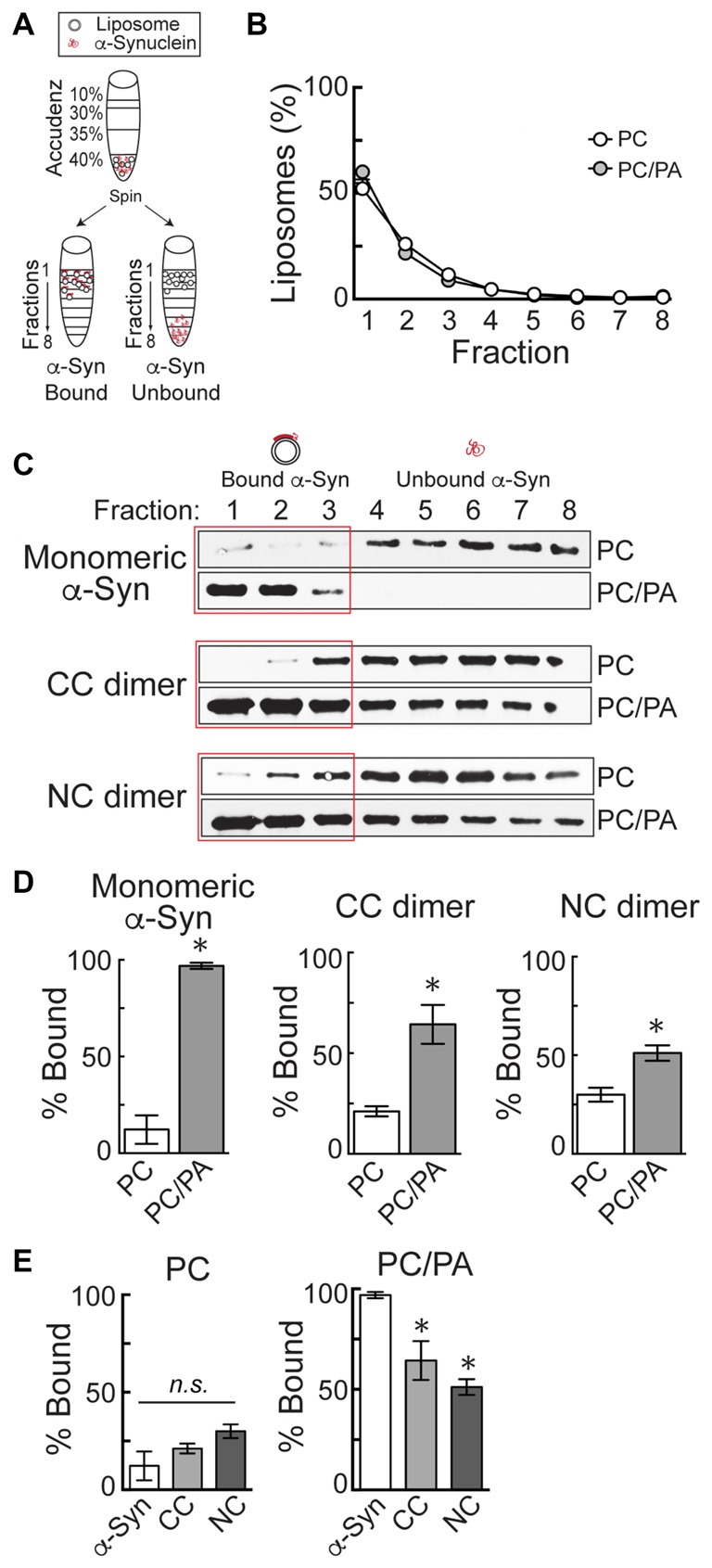
α-Synuclein dimers bind to phosphatidic acid (PA)-containing liposomes. **(A)** Diagram of the liposome binding assay. After incubation of α-synuclein with liposomes, ultracentrifugation in an Accudenz gradient results in flotation of liposomes along with any bound protein to top fractions. **(B)** Fluorometer measurements showing the distribution of liposomes after ultracentrifugation. Note that >90% of liposomes are found in fractions 1–3. **(C)** Western blots showing the amount of α-synuclein that is bound to liposomes (fractions 1–3) or unbound (fractions 4–8). Monomeric α-synuclein exhibits very little binding to phosphatidylcholine (PC) liposomes, but binds robustly to 1:1 PC/PA liposomes. CC and NC dimers follow a similar pattern of binding. **(D)** Quantification of band intensities from Western blots. Monomeric and dimeric α-synuclein all exhibit a significant increase in binding to PC/PA liposomes, compared to PC alone. Bars represent mean ± SEM of the percentage of total α-synuclein bound, as measured in *n* = 3–4 independent experiments. Asterisks indicate statistical significance by Student’s *t*-test (*p* < 0.05). **(E)** Direct comparison of PC and PC/PA binding by monomeric or dimeric α-synuclein. Asterisks indicate statistical significant by analysis of variance (ANOVA; *p* < 0.05).

Liposomes were generated using 16:0–18:1 phosphatidylcholine (PC), or alternatively 16:0–18:1 1-phosphatidic acid (PA) and PC in a 1:1 ratio (Avanti Polar Lipids, Alabaster, AL, USA). The PC component of all liposomes included 1% fluorescently tagged 18:1–12:0 nitrobenzoxadiazole-PC (NBD-PC). Liposomes were prepared as previously described (Busch et al., 2014). Briefly, 1 mg of total lipids was added to 200 μl of 2:1 chloroform:methanol and dried over a stream of nitrogen. The membranes were swollen in 300 mM sucrose at 37°C for 20 min and then vortexed, thus forming large, heterogeneous liposomes. Liposomes were sonicated in order to generate small, uniform liposomes (~30 nm diameter) and then ultracentrifuged at 80,000× *g* for 20 min at 25°C to remove any large liposomes by sedimentation. Liposome binding was performed as previously described (Burré et al., 2012; Busch et al., 2014). The small, sonicated liposomes (17 μl) were incubated with 5 μg of α-synuclein in HKE buffer (25 mM HEPES, pH 7.4, 150 mM KCl, 1 mM EDTA) for 2 h at room temperature (RT; 100 μl total volume). Samples were then loaded onto the bottom of an Accudenz gradient (40%, 35%, 30%, 0% from bottom to top; 800 μl total; Accurate Chemical and Scientific Corp., Westbury, NY, USA) and ultracentrifuged at 280,000× *g* for 3 h at RT (Figure 2A). After ultracentrifugation, samples were separated into eight equal fractions, and fluorescence measurements were made on each fraction using a Nanodrop 3300 in order to determine the distribution of liposomes. In all experiments, it was determined that >90% of the liposomes floated up into fractions 1–3 (Figure 2B). The amount of α-synuclein in each fraction was subsequently determined by Western blotting, as in prior studies (Figure 2C; Burré et al., 2012; Busch et al., 2014). α-Synuclein was detected using a pan-synuclein antibody raised against the N-terminus of human α-synuclein, which recognized both monomeric and dimeric α-synuclein (1:1000; ab53726; Abcam, Cambridge, MA, USA). The secondary antibody used was a goat anti-rabbit HRP conjugated IgG (H + L) (Thermo Scientific, Waltham, MA, USA). Protein bands were detected using Pierce™ ECL Western blotting substrate (Thermo Scientific, Waltham, MA, USA). α-Synuclein band intensity was quantified in each fraction using FIJI 2.0.0 software, and the percentage of liposome-bound α-synuclein in fractions 1–3 was determined in *n* = 3–4 independent experiments for each experimental condition.

### Microinjection, Stimulation and EM Imaging at Lamprey Synapses

**Figure 3:**
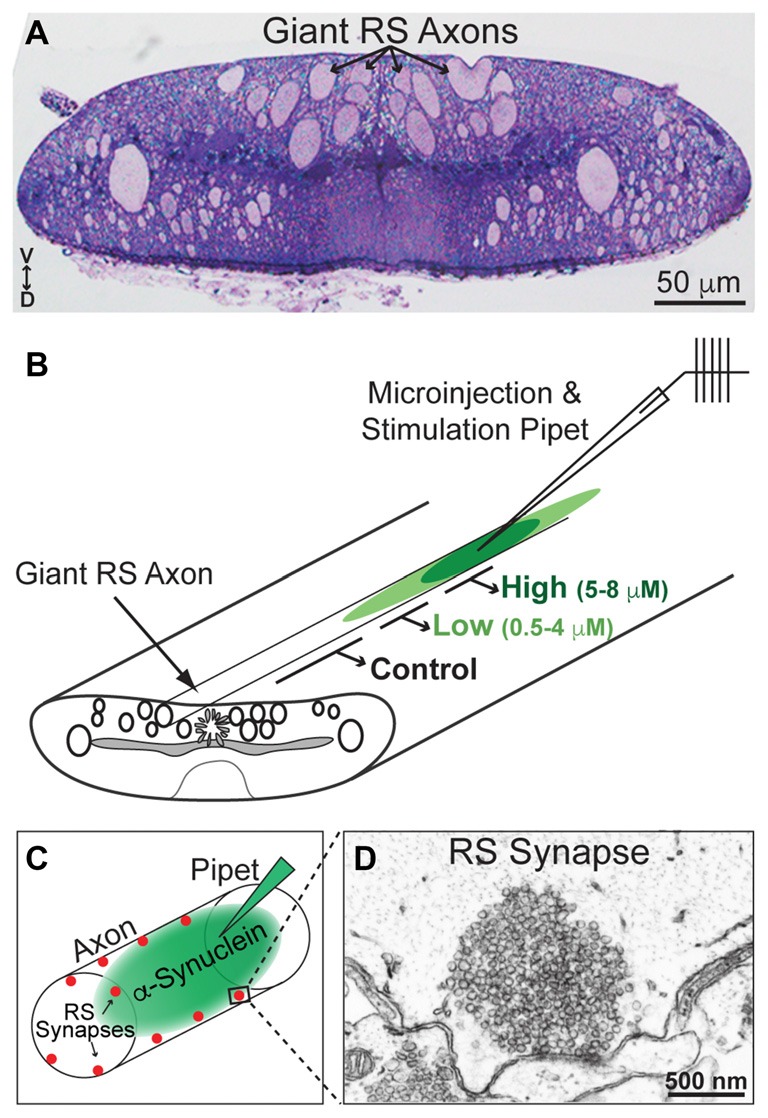
Lamprey reticulospinal (RS) synapses and injection strategy. **(A)** Cross-section (1 μm thick) of a lamprey spinal cord stained with toluidine blue. Note the giant RS axons in the ventromedial tract. V—ventral; D—Dorsal. **(B)** Diagram of a lamprey spinal cord showing the strategy for axonal microinjection and stimulation. After microinjection of the α-synuclein dimers, the synapses within three regions of the axon were evaluated: those treated with higher concentrations of the protein (5–8 μM), lower concentrations of the protein (0.5–4 μM), or no protein (0 μM; Controls). These regions were determined based on diffusion of a co-injected fluorescent dye. **(C)** Diagram of an injected RS axon indicating the locations of giant RS synapses along the axonal perimeter. **(D)** Electron micrograph of an unstimulated, control RS synapse showing the large size of the synaptic vesicle (SV) cluster.

All animal procedures were approved by the Institutional Animal Care and Use Committee at the Marine Biological Laboratory in Woods Hole, MA and in accordance with standards set by the National Institutes of Health. Lampreys (*Petromyzon marinus*; 11–13 cm) were anesthetized in MS-222 (0.1 g/L; Western Chemicals Inc., Ferndale, WA, USA), after which 2–3 cm pieces of spinal cords were microdissected and pinned in a Sylgard-lined petri dish containing fresh, oxygenated Lamprey Ringer (100 mM NaCl, 2.1 mM KCl, 1.8 mM MgCl_2_, 4 mM glucose, 2 mM HEPES, 0.5 mM L-glutamine, 2.6 mM CaCl_2,_ pH 7.4). CC dimer (50 μM), NC dimer (80 μM), Dynasore (80 μM; Abcam ab120192; Cambridge, MA, USA) and monomeric α-synuclein (160 μM; rPeptide), all diluted in lamprey internal solution to the indicated stock concentrations, were loaded into glass microelectrodes and subsequently injected into lamprey giant RS axons using small pulses of nitrogen delivered via a Toohey Spritzer. All reagents were co-injected with a fluorescent dextran approximating their molecular weights in order to determine how much reagent was injected and how far it spread via axonal diffusion. For experiments with α-synuclein dimers, we co-injected a 40 kDa fluorescein dextran (Thermo Fisher Scientific, D1845; Waltham, MA, USA). For Dynasore experiments, we co-injected a 3 kDa fluorescein dextran (Thermo Fisher Scientific, D3309; Waltham, MA, USA). For experiments with monomeric α-synuclein, we co-injected a 10 kDa Alexa Fluor^®^ 488 dextran (Thermo Fisher Scientific, D22910; Waltham, MA, USA). The spread of the fluorescent dye, as well as its intensity along the axon relative to the injection site, were monitored during the injection in order to estimate the final intra-axonal concentration of the proteins (see Figure 3B). By the fluorescence measurements, the α-synuclein and other reagents were diluted >10–20 times from the stock concentration in typical injection experiments.

After the injections were complete, the lamprey giant RS axons were stimulated intracellularly with short current pulses (30–70 nA; 1 ms) in order to trigger action potentials (20 Hz, 5 min). Spinal cords were fixed immediately in 3% glutaraldehyde, 2% paraformaldehyde in 0.1 M Na cacodylate, pH 7.4, processed with 2% osmium in 2% potassium ferrocyanide (EM Sciences, Hatfield, PA, USA), stained *en bloc* with 2% uranyl acetate and embedded in EMbed 812 resin, as previously described (Morgan et al., 2004, 2013b; Busch et al., 2014). In order to evaluate the tissue quality and to identify the injected axon, histological staining was performed on 1 μm sections stained with a 1% toluidine blue, 1% sodium borate solution for 1–2 min (see Figure 3A, for example). For electron microscopy, spinal cords were thin sectioned at 70 nm onto formvar coated copper slot grids, post stained with 2% uranyl acetate and 0.4% lead citrate, and subsequently imaged using a JEOL JEM 200CX transmission electron microscope at 37,000× or 59,000× magnification. For each experimental condition, all synapses containing a clearly defined presynaptic active zone were imaged in series. Images that contained the center of the active zone were selected for further morphometric analyses (*n* = 10–26 synapses per condition).

As described previously, morphometric analyses were performed on all synaptic membranes within a 1 μm radius of the presynaptic active zone using FIJI 2.0.0 by a researcher blinded to the experimental conditions (Morgan et al., 2004, 2013b; Busch et al., 2014). These analyses included the number of SVs, size of plasma membrane (PM) evaginations, number and size of membranous “cisternae”, and number and stage of CCPs and CCVs per synapse, as determined from single thin sections. SVs were defined as small, clear round vesicles <100 nm in diameter. PM evaginations were measured by drawing a straight, 1 μm line from the edge of the active zone to the nearest point on the axolemma in either direction, measuring the curved distance between these points, then recording the average from each synapse. “Cisternae” were defined as larger, atypical vesicles >100 nm in diameter. CCP/Vs were determined by the presence of an electron dense coat around the vesicle membrane. In addition, a total membrane analysis was performed on each synapse in order to determine how the synaptic membranes were redistributed after acute perturbations. Here, the SV and CCP/V membrane areas were determined by multiplying the surface area of a sphere (4πr^2^) by the number of SVs or CCP/Vs at each synapse, respectively, where *r* = *d*/2 and *d* = the average diameter of 200 SVs measured from each experimental condition. Cisternae and PM measurements were obtained by multiplying the summed cisternae perimeters and length of PM evaginations by the section thickness (70 nm) in order to obtain the total amount of membrane area. For the analysis on the necks of CCPs (Figure 6), the neck width was measured at its narrowest point, and the neck length was measured from the PM to the beginning of the clathrin coat. All graphed data represent the mean ± SEM per section per synapse. Graphs were generated using Origin Pro 7.0 (OriginLab, Northampton, MA, USA). As previously described (Busch et al., 2014), 3D reconstructions of five serial electron micrographs were generated using Reconstruct software version 1.1.0.0 (Fiala, 2005). PM and cisternae were rendered as trace slabs; SVs and CCP/Vs were rendered as 50 nm and 90 nm spheres, respectively; active zone was rendered as a Boissonnat surface.

**Figure 4:**
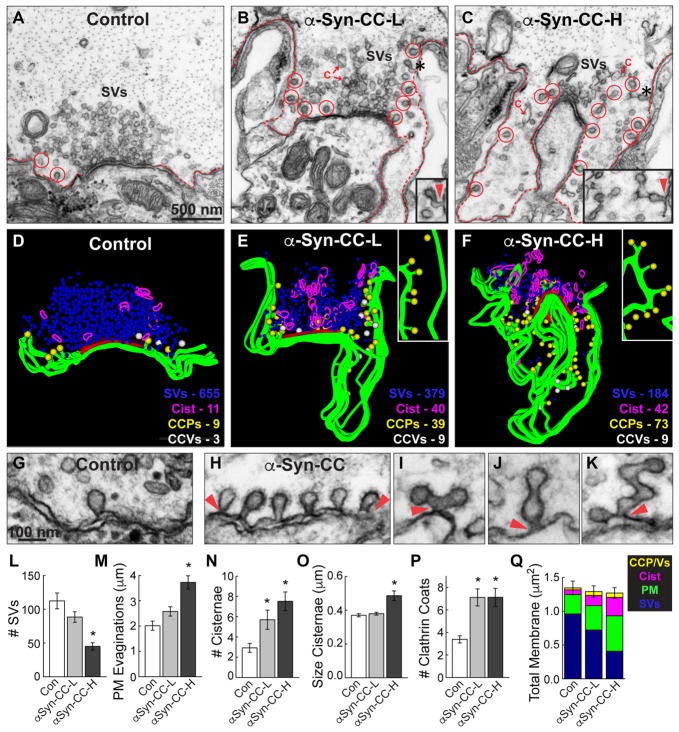
α-Synuclein CC dimer inhibits SV endocytosis. **(A–C)** Electron micrographs showing a typical control synapse, compared to synapses treated with either low or high concentrations of α-synuclein CC dimer (α-Syn-CC). L, low concentration; H, high concentration. Synapses treated with CC dimer exhibited SV recycling defects, as shown by a reduction in SVs, expanded plasma membrane (PM) evaginations (dotted lines), and greater numbers of endocytic intermediates including cisternae (C) and clathrin-coated pits (CCPs) with constricted necks (red circles). Insets show longer, branched tubules ending in clathrin coats. Arrowheads indicate their connections with the PM. Asterisks mark the complex budding structures shown in the insets. Scale bar in **(A)** also applies to **(B,C)**. **(D–F)** 3D reconstructions reveal the extent of the endocytic defects induced by CC dimer, shown by a loss of SVs (blue), larger PM evaginations (green) and build up of cisternae (magenta), CCPs (yellow) and CCVs (white). Red slab marks the active zone. **(G–K)** Electron micrographs show typical CCPs at control **(G)** and CC dimer-treated synapses **(H–K)**. Note the abundance of CCPs, including those with complex structures induced by CC dimer. Arrowheads indicate their connections with the PM. Scale bar in **(G)** applies to **(H–K)**. **(L–Q)** Quantification of the SV recycling defects. The reduction in SVs **(L)** was compensated by increased PM evaginations, cisternae and CCPs and vesicles **(M–Q)**. Data reported indicate the averages per synapse, as measured from single sections. Bars represent mean ± SEM from *n* = 26–27 synapses, 2–3 axons. Asterisks indicate statistical significance (*p* < 0.05) by ANOVA, as compared to controls.

**Figure 5:**
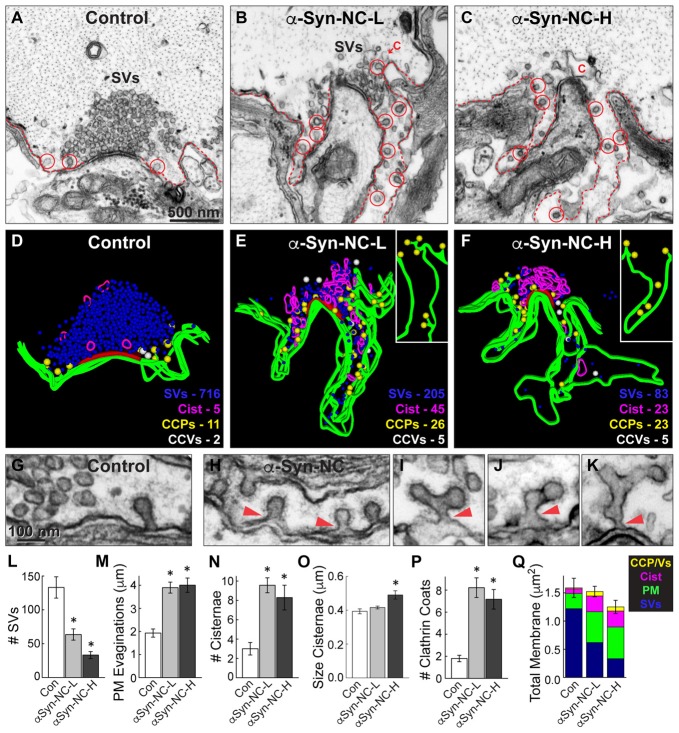
α-Synuclein NC dimer also inhibits SV endocytosis. **(A–C)** Electron micrographs showing a control synapse and those treated with either low or high concentrations of α-synuclein NC dimer (α-Syn-NC). L, low concentration; H, high concentration. NC dimer produced a phenotype that nearly identical to that caused by CC dimer (see Figure 4). After treatment with NC dimer, synapses exhibited SV recycling defects, as shown by a reduction in SVs, expanded PM evaginations (dotted lines) and greater numbers of cisternae (C) and CCPs with constricted necks (red circles). Scale bar in **(A)** applies to **(B,C)**. **(D–F)** 3D reconstructions show the SV recycling defects induced by NC dimer, as demonstrated by a loss of SVs (blue), larger PM evaginations (green) and build up of cisternae (magenta), CCPs (yellow) and CCVs (white). Red slab marks the active zone. **(G–K)** Electron micrographs show typical CCPs at control **(G)** and NC dimer-treated synapses **(H–K)**. Note the abundance of CCPs, including those with complex structures induced by NC dimer. Arrowheads indicate their connections with the PM. **(L–Q)** Quantification of the SV recycling defects produced by NC dimer. The reduction in SVs **(L)** was compensated by increased PM evaginations, cisternae and CCPs and vesicles **(M–Q)**. Data reported indicate the averages per synapse, as measured from single sections. Bars represent mean ± SEM from *n* = 10–24 synapses, 1–2 axons. Asterisks indicate statistical significance (*p* < 0.05) by ANOVA, as compared to the controls.

**Figure 6:**
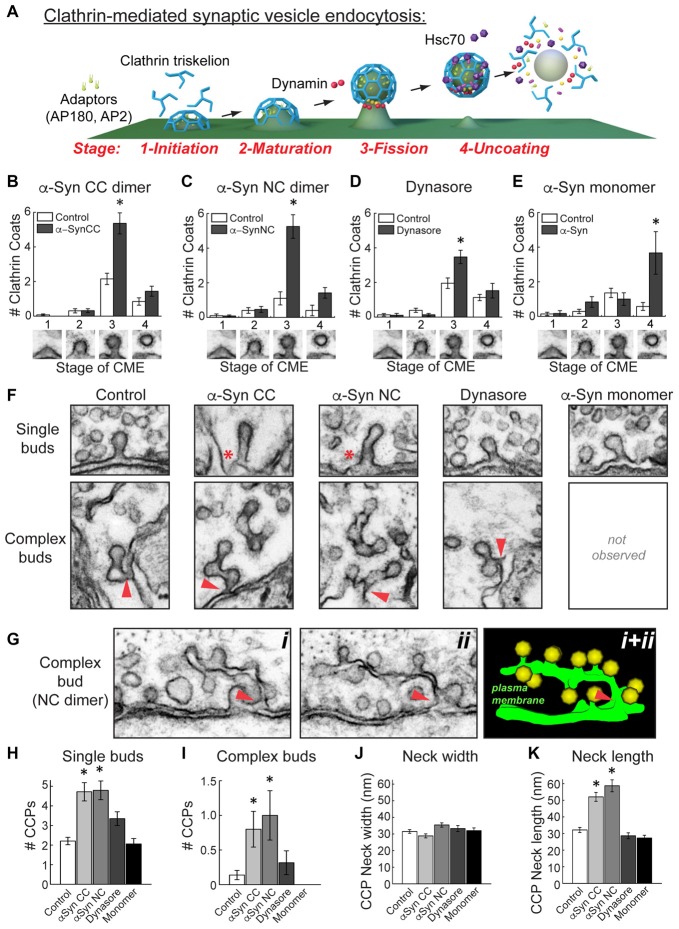
α-Synuclein dimers inhibit vesicle fission and induce membrane tubulation during clathrin-mediated SV endocytosis. **(A)** Model of clathrin-mediated SV endocytosis, including several molecular players. Graphics generated by Jack Cook (Woods Hole Oceanographic Institution) using Cinema 4D. **(B–E)** Quantitative analysis of each morphologically distinct stage of CCP and CCV formation. Refer to **(A)** for individual stages. This analysis revealed that CC and NC dimer inhibited vesicle fission, as indicated by a selective increase in stage 3 CCPs. Similarly, and as expected, Dynasore inhibited vesicle fission. In contrast, monomeric α-synuclein inhibited a different stage of clathrin-mediated endocytosis, during clathrin uncoating (stage 4). Data reported indicate the averages per synapse, as measured from single sections. Bars represent mean ± SEM from *n* = 10–26 synapses, 1–3 axons. Asterisks indicate statistical significance (*p* < 0.05) by ANOVA, as compared to the controls. **(F)** Electron micrographs showing the observed morphologies of CCPs under the various experimental conditions. (Top) CC and NC dimers induced individual CCPs with longer necks (asterisks), compared to control, Dynasore, or monomer-treated synapses. (Bottom) CC and NC dimers also induced more complex budding structures extending from branched membrane tubules. Arrowheads indicate their connections with the PM. **(G)** Serial images (*i, ii*) and a 3D reconstruction (*i + ii*) showing a complex tubule extending from the PM ending in multiple CCPs. Arrowheads indicate the connections with the PM. **(H–K)** The α-synuclein dimers significantly increased the number of single and complex buds. In addition, neck length on CCPs, but not neck width, was increased with the α-synuclein dimers. Data reported indicate the averages per synapse, as measured from single sections. Bars represent mean ± SEM from *n* = 6–28 synapses, 1–3 axons. Asterisks indicate statistical significance (*p* < 0.05) by ANOVA, as compared to the controls.

## Results

### Stable, Recombinant α-Synuclein Dimers

Human α-synuclein (140 a.a) comprises an N-terminal domain that mediates membrane binding and folds into an amphipathic helix upon interaction with small, highly curved vesicles; a central core non-amyloid component (NAC) domain that mediates self-association; and a less structured C-terminus containing acidic residues and prolines (Figure 1A). The goal of this study was to determine how α-synuclein dimers affect SV trafficking. We therefore synthesized two stable recombinant α-synuclein dimers, as described in the “Materials and Methods” section and a prior study (Pivato et al., 2012). CC dimer was generated by covalent linkage of two α-synuclein molecules at their C-termini (Figure 1B). NC dimer was generated by expression of two α-synuclein molecules in tandem in a single polypeptide, thereby bringing together the N- terminus of one α-synuclein with the C-terminus of the other (Figure 1B). While the exact conformations of endogenous α-synuclein dimers are unknown, formation of parallel and anti-parallel multimers was implied through the use of bimolecular fluorescence complementation assays (Wang et al., 2014). Thus, the recombinant α-synuclein dimers, which likely adopt parallel and anti-parallel conformations, allowed us to test the efficacy of each form. The α-synuclein dimers were stable in SDS-PAGE under non-reducing conditions (Figure 1C). While monomeric α-synuclein ran at ~14 kDa by SDS-PAGE, CC and NC dimers ran higher around ~28 kDa, as expected for these larger proteins (Figure 1C).

### α-Synuclein Dimers Bind to Small Liposomes Containing Acidic Phospholipids

A prior study showed that the α-synuclein CC and NC dimers exhibited many biochemical properties similar to monomeric α-synuclein, including folding, fibrillation and aggregation, albeit with slightly altered dynamics (Pivato et al., 2012). Here, we additionally characterized the lipid binding capabilities of the α-synuclein dimers. Monomeric α-synuclein binds *in vitro* to anionic phospholipids when presented in small, highly-curved liposomes (30–50 nm diameter; Davidson et al., 1998; Burré et al., 2010, 2012, 2015; Busch et al., 2014). Lipid binding induces the N-terminal domain of α-synuclein to adopt an alpha helical conformation, which likely contributes to its ability to bind to SVs *in vivo* (Maroteaux et al., 1988; Davidson et al., 1998; Fortin et al., 2005). We previously showed that α-synuclein point mutations that reduced alpha helical content and liposome binding also greatly reduced the synuclein-induced vesicle trafficking defects at lamprey RS synapses, implicating membrane binding as a significant contributor to the observed synaptic defects (Busch et al., 2014). These findings are consistent with studies at mammalian synapses (Nemani et al., 2010; Xu et al., 2016; Eguchi et al., 2017).

To estimate whether α-synuclein dimers might also induce vesicle trafficking defects, we therefore tested the lipid binding capacity of the α-synuclein dimers using a well-established liposome binding assay (Figure 2; Burré et al., 2010, 2012; Busch et al., 2014). Briefly, monomeric or dimeric α-synuclein was incubated with small, sonicated liposomes (~30 nm) and then loaded onto the bottom of an Accudenz gradient. After ultracentrifugation and separation into eight fractions (Figure 2A), fluorometer measurements indicated that 90%–95% of the liposomes floated into the top three fractions (Figure 2B). Figure 2B shows representative fluorometer data obtained from liposomes made with PC or a 1:1 mixture of PC and PA. Consequently, any liposome-bound α-synuclein, as detected by Western blotting, will also be present in fractions 1–3, while unbound α-synuclein will remain in fractions 4–8 (Figure 2A). As previously reported (Burré et al., 2012; Busch et al., 2014), monomeric α-synuclein exhibited very little binding to PC liposomes, but bound robustly to PC/PA liposomes (Figures 2C,D; *Monomeric* α*-synuclein—*PC: 12.3 ± 7.4%, *n* = 4; PC/PA: 96.9 ± 1.6%, *n* = 3; Student’s *t*-test, *p* = 0.0002). Similarly, CC and NC dimers exhibited stronger binding to PC/PA liposomes than to PC only liposomes, as shown by a shift of the protein into the top fractions of the column (Figures 2C,D). Quantification of Western blot band intensities revealed significantly greater binding of the α-synuclein dimers to PC/PA liposomes, compared to liposomes containing only PC (Figure 2D; *CC dimer—*PC: 21.2 ± 2.5%, *n* = 3; PC/PA: 64.4 ± 9.7%, *n* = 3; Student’s *t*-test, *p* = 0.01; *NC dimer—*PC: 30.0 ± 3.5%, *n* = 3; PC/PA: 51.1 ± 3.9%, *n* = 3; Student’s *t*-test, *p* = 0.02). When directly compared, α-synuclein, CC and NC dimers exhibited no difference in binding to PC liposomes (ANOVA *p* > 0.05; Figure 2E). However, CC and NC dimers did exhibit a 34% and 47% reduction in binding to PC/PA liposomes, respectively (ANOVA *p* < 0.05; Figure 2E). Thus, while α-synuclein dimers preferentially bind to liposomes containing an anionic phospholipid such as PA, they also bind with reduced efficacy compared to monomeric α-synuclein, predicting that they may produce distinct effects on SV trafficking.

### α-Synuclein Dimers Inhibit Synaptic Vesicle Recycling, Including Effects on the Clathrin Pathway

Next, we wanted to determine whether and how acute introduction of α-synuclein dimers affects SV trafficking. We used as our experimental model lamprey giant RS synapses, which are large (1–2 μm in diameter) glutamatergic, *en passant* synapses that reside along the perimeter of the giant axons (20–40 μm in diameter) within the ventromedial spinal cord (Figure 3A). α-Synuclein CC and NC dimers were acutely delivered via microinjection into the giant RS axons, providing direct access of the proteins to the presynapses (Figures 3B,C). Co-injection of a fluorescent dye with a similar molecular weight allowed us to approximate the concentrations of the α-synuclein dimers along the axon with respect to the injection site (Figure 3B). After dimer injections, the axons were stimulated intracellularly with action potentials (20 Hz, 5 min). Immediately following the stimulation period, spinal cords were fixed and processed for electron microscopy for visualization of RS synapses. The RS synapses consist of large vesicle clusters (~100–150 SVs/section; 1000–2000 SVs total) positioned along the axolemma and centered around an electron dense active zone (Figures 3C,D). We examined the ultrastructural effects of CC and NC dimers on synapses located within three regions of the axon (Figure 3B): (1) 25–225 μm from the injection site where α-synuclein dimer concentrations were higher (estimated 5–8 μM; see “Materials and Methods” section); (2) 250–390 μm from the injection site where α-synuclein dimer concentrations were lower (estimated 0.5–4 μM); and (3) >400 μm from the injection site where no exogenous α-synuclein was present (0 μM). This last region allowed us to assess the normal morphologies of untreated, stimulated synapses, thereby providing an internal control. The maximal amount of exogenous α-synuclein injected (5–8 μM) was only 2–3 times greater than the current estimate of endogenous α-synuclein at synapses, which is 3–6 μM (Westphal and Chandra, 2013). Thus, the additional α-synuclein introduced to synapses is commensurate with the two- to three-fold overexpression levels observed in PD and animal models (Singleton et al., 2003; Rockenstein et al., 2005; Nemani et al., 2010; Scott et al., 2010).

Under these stimulation conditions, control RS synapses exhibited large SV clusters, shallow PM evaginations and only a few CCPs (Figures 4A,D). Compared to unstimulated synapses (Figure 3D), stimulated synapses have slightly fewer vesicles, less compact SV clusters and more CCPs, indicating that vesicle recycling is occurring. In comparison, at synapses treated with low concentrations of α-synuclein CC dimer (α-Syn-CC-L), the SV clusters were even smaller; PM evaginations were more extended; and, large vesicles (>100 nm diameter) with irregular shapes, which we call “cisternae,” were more abundant (Figures 4B,E). In addition, there were greater numbers of clathrin-coated pits (CCPs), including complex tubules ending in clathrin coats, budding from the PM (Figures 4B,E; see insets). A loss of SVs combined with an increase in PM and endocytic intermediates is consistent with an inhibition of SV endocytosis from the PM. At higher concentrations of CC dimer (α-Syn-CC-H), a similar phenotype was observed with fewer SVs, larger PM evaginations and more cisternae and CCPs, but the effects were even more pronounced (Figures 4C,F). 3D reconstructions of serial sections corroborated the endocytic phenotypes observed in single sections and also further emphasized the build up of CCPs at the PM (Figures 4D–F; yellow spheres and insets). In contrast, the free CCVs did not appear to be dramatically affected (Figures 4E,F, white spheres). A higher magnification view of the synaptic membranes revealed that control synapses typically exhibited only one or several CCPs with constricted necks (Figure 4G). In comparison, synapses treated with CC dimer often exhibited rows of constricted CCPs with normal appearance emanating directly from the PM (Figure 4H, arrowheads), as well as atypical complex buds and tubules ending in multiple clathrin coats (Figures 4I–K, arrowheads), suggesting a defect in vesicle fission during SV endocytosis.

Morphometric analyses further corroborated that α-synuclein CC dimer induced endocytic defects at synapses. The number of SVs was significantly reduced at synapses treated with CC dimer, compared to controls, especially at higher concentrations (Figure 4L; Control: 112.4 ± 11.7 SVs, *n* = 26 synapses, 3 axons; α-Syn-CC-L: 88.5 ± 7.8 SVs, *n* = 27 synapses, 2 axons; α-Syn-CC-H: 45 ± 5.3 SVs, *n* = 25 synapses, 2 axons; ANOVA *p* = 3.3 × 10^−6^; Tukey’s *post hoc*). The mean diameter of the remaining SVs was not significantly altered (Control: 52.2 ± 0.7 nm, *n* = 200 SVs; α-Syn-CC-L: 50.9 ± 0.6 nm, *n* = 200 SVs; α-Syn-CC-H: 53.7 ± 0.9 nm, *n* = 200 SVs; ANOVA *p* = 0.03; Tukey’s *post hoc*). PM evaginations became progressively larger with increased concentrations of CC dimer (Figure 4M; Control: 2.0 ± 0.2 μm, *n* = 26 synapses, 3 axons; α-Syn-CC-L: 2.6 ± 0.2 μm, *n* = 27 synapses, 2 axons; α-Syn-CC-H: 3.7 ± 0.3 μm, *n* = 25 synapses, 2 axons; ANOVA *p* = 9.7 × 10^−7^, Tukey’s *post hoc*). Both the number and size of cisternae were also significantly increased after treatment with CC dimer (Figures 4N,O) *(# Cisternae—*Control: 2.9 ± 0.4 cisternae, *n* = 26 synapses, 3 axons; α-Syn-CC-L: 5.7 ± 1.0 cisternae, *n* = 27 synapses, 2 axons; α-Syn-CC-H: 7.5 ± 0.9 cisternae, *n* = 25 synapses, 2 axons; ANOVA *p* = 6.5 × 10^−4^, Tukey’s *post hoc*; *Size Cisternae—*Control: 0.37 ± 0.01 μm, *n* = 76 cisternae, 26 synapses; α-Syn-CC-L: 0.38 ± 0.01 μm, *n* = 153 cisternae, 27 synapses; α-Syn-CC-H: 0.49 ± 0.03 μm, *n* = 186 cisternae, 25 synapses; ANOVA *p* = 2.3 × 10^−4^, Tukey’s *post hoc*). In addition, the total number of CCPs and CCVs combined (i.e., “clathrin coats”) was also significantly increased (Figure 4P; Control: 3.4 ± 0.3 coats, *n* = 26 synapses, 3 axons; α-Syn-CC-L: 7.1 ± 0.8 coats, *n* = 27 synapses, 2 axons; α-Syn-CC-H: 7.1 ± 0.8 coats, *n* = 25 synapses, 2 axons; ANOVA *p* = 0, Tukey’s *post hoc*). We also performed a comprehensive analysis of the total membrane distribution at synapses. This analysis revealed that the loss of SV membrane area was compensated by greater membrane area in the PM, cisternae and clathrin coat compartments (Figure 4Q; Control: 1.4 ± 0.1 μm^2^, *n* = 26 synapses, 3 axons; α-Syn-CC-L: 1.3 ± 0.08 μm^2^, *n* = 27 synapses, 2 axons; α-Syn-CC-H: 1.3 ± 0.08 μm^2^, *n* = 25 synapses, 2 axons; ANOVA *p* = 0.84, Tukey’s *post hoc*). Taken together, these data indicate that α-synuclein CC dimer induced a dose-dependent inhibition of SV endocytosis from the PM, including effects on the clathrin pathway. The atypical CCPs observed further suggested that CC dimer impairs vesicle fission during clathrin-mediated endocytosis.

Similarly, we also examined the synaptic effects produced by NC dimer. Acute introduction of α-synuclein NC dimer produced nearly identical effects on SV endocytosis, but the effects were slightly more pronounced even at the lower concentrations. Both low and high concentrations of NC dimer impaired synaptic morphology, resulting in progressively smaller SV clusters (Figures 5A–F,L; Control: 133.1 ± 15.8 SVs, *n* = 10 synapses, 2 axons; α-Syn-NC-L: 63.5 ± 8.3 SVs, *n* = 24 synapses, 1 axon; α-Syn-NC-H: 33.2 ± 5.2 SVs, *n* = 23 synapses, 1 axon; ANOVA *p* = 9.96 × 10^−9^; Tukey’s *post hoc*). At the higher concentrations of NC dimer, the mean SV diameter was slightly larger than at control synapses (Control: 54.0 ± 0.6 nm, *n* = 200 SVs, 10 synapses; α-Syn-NC-L: 55.7 ± 0.6 nm, *n* = 200 SVs, 10 synapses; α-Syn-NC-H: 56.5 ± 0.8 nm, *n* = 200 SVs, 10 synapses; ANOVA *p* = 0.02; Tukey’s *post hoc*). PM evaginations were significantly larger after treatment with NC dimer (Figures 5A–F,M; Control: 1.9 ± 0.2 μm, *n* = 10 synapses, 2 axons; α-Syn-NC-L: 4.0 ± 0.3 μm, *n* = 24 synapses, 1 axon; α-Syn-NC-H: 4.0 ± 0.4 μm, *n* = 23 synapses, 1 axon; ANOVA *p* = 0.00011, Tukey’s *post hoc*). As with CC dimer, the number and size of cisternae were also significantly increased (Figures 5N,O; *# Cisternae—*Control: 3.0 ± 0.6 cisternae, *n* = 10 synapses, 2 axons; α-Syn-NC-L: 9.6 ± 0.8 cisternae, *n* = 24 synapses, 1 axon; α-Syn-CC-H: 8.3 ± 1.3 cisternae, *n* = 23 synapses, 1 axon; ANOVA *p* = 0.0026, Tukey’s *post hoc*; *Size Cisternae—*Control: 0.39 ± 0.01 μm, *n* = 30 cisternae, 10 synapses; α-Syn-NC-L: 0.42 ± 0.01 μm, *n* = 219 cisternae, 24 synapses; α-Syn-NC-H: 0.49 ± 0.02 μm, *n* = 188 cisternae, 23 synapses; ANOVA *p* = 0.0039, Tukey’s *post hoc*). As with CC dimer, NC dimer also induced aberrant CCP budding from the PM (Figures 5E–K), resulting in greater numbers of CCPs with normal appearance (Figure 5H) as well as atypical, complex budding structures extending from the PM (Figures 5I–K, arrowheads). The total number of clathrin coats was significantly greater than at control synapses (Figure 5P; Control: 1.8 ± 0.3 coats, *n* = 10 synapses, 2 axons; α-Syn-NC-L: 8.2 ± 0.9 coats, *n* = 24 synapses, 1 axon; α-Syn-NC-H: 7.2 ± 0.9 coats, *n* = 23 synapses, 1 axon; ANOVA *p* = 2.81 × 10^−4^, Tukey’s *post hoc*). Total membrane analysis revealed that the loss of SV membrane area induced by NC dimer was compensated by greater membrane area in the PM, cisternae, and CCPs and CCVs (Figure 5Q; Control: 1.57 ± 0.17 μm^2^, *n* = 10 synapses, 1 axon; α-Syn-NC-L: 1.44 ± 0.09 μm^2^, *n* = 23 synapses, 1 axon; α-Syn-NC-H: 1.25 ± 0.12 μm^2^, *n* = 24 synapses, 1 axon; ANOVA *p* = 0.21, Tukey’s *post hoc*). Thus, as with CC dimer, NC dimer also impaired clathrin-mediated SV endocytosis with apparent effects on the vesicle fission process.

### α-Synuclein Dimers Inhibit Vesicle Fission during Clathrin-Mediated Endocytosis

Clathrin-mediated SV endocytosis proceeds through several morphologically distinct stages (Figure 6A; Brodin et al., 2000; Morgan et al., 2002, 2013a; Saheki and De Camilli, 2012). As in our prior studies, we have defined them according to the stages of clathrin coat formation (Morgan et al., 2004, 2013b; Bourne et al., 2006; Busch et al., 2014). Initiation of clathrin coat formation begins when the adaptor proteins AP180 and AP2 recruit clathrin triskelions to the PM (Stage 1). Following maturation of the clathrin coat (Stage 2), the GTPase dynamin is recruited to the neck of the CCP, where its GTPase activity leads to constriction and vesicle fission (Stage 3). Once separated from the PM, the free CCV is uncoated by the ATP activity of the chaperone protein Hsc70 (Stage 4). The aberrant vesicle budding observed with CC and NC dimers suggested that they may be affecting the process of vesicle budding at the fission step. To test this, we examined the number of CCPs (Stage 1–3) and CCVs (Stage 4) at synapses after treatment with the α-synuclein dimers. Both CC and NC dimers selectively and significantly increased the number of Stage 3 CCPs with constricted necks, indicating an impairment in vesicle fission (Figures 6B,C; CC dimer: *Stage 1*-Control: 0.1 ± 0.1 CCPs; α-Syn-CC-H: 0 ± 0 CCPs; *Stage 2*-Control: 0.3 ± 0.1 CCPs; α-Syn-CC-H: 0.3 ± 0.1 CCPs; *Stage 3*-Control: 2.2 ± 0.3 CCPs; α-Syn-CC-H: 5.4 ± 0.6 CCPs; *Stage 4*-Control: 0.9 ± 0.2 CCVs; α-Syn-CC-H: 1.4 ± 0.3 CCVs; *n* = 10–26 synapses per condition; ANOVA *p* < 0.00005, Tukey’s *post hoc*; NC dimer: *Stage 1*-Control: 0.1 ± 0.1 CCPs; α-Syn-NC-H: 0.1 ± 0.1 CCPs; *Stage 2*-Control: 0.4 ± 0.2 CCPs; α-Syn-NC-H: 0.5 ± 0.2 CCPs; *Stage 3*-Control: 1.1 ± 0.4 CCPs; α-Syn-NC-H: 5.3 ± 0.7 CCPs; *Stage 4*-Control: 0.4 ± 0.3 CCVs; α-Syn-CC-H: 1.4 ± 0.3 CCVs; *n* = 10–26 synapses per condition; ANOVA *p* = 0, Tukey’s *post hoc*).

To corroborate that α-synuclein dimers were indeed causing a bona fide fission defect, we also examined the effects of Dynasore (80 μM), a potent and selective inhibitor of dynamin GTPase activity that arrests late stages of budding prior to vesicle fission, leading to an increase in CCPs with constricted necks that are still attached to the membrane (Macia et al., 2006; Newton et al., 2006). We performed these experiments using the same injection and stimulation conditions as in the α-synuclein dimer experiments. After diffusion and dilution, we estimate that the final axonal concentration of Dynasore was ~4–8 μM. As with CC and NC dimers, Dynasore also selectively increased the number of stage 3 CCPs (Figure 6D; *Stage 1*-Control: 0.1 ± 0.1 CCPs; Dynasore: 0.1 ± 0.1 CCPs; *Stage 2*-Control: 0.4 ± 0.1 CCPs; Dynasore: 0.1 ± 0.1 CCPs; *Stage 3*-Control: 2.0 ± 0.2 CCPs; Dynasore: 3.5 ± 0.4 CCPs; *Stage 4*-Control: 1.1 ± 0.2 CCVs; Dynasore: 1.5 ± 0.4 CCVs; *n* = 23 control synapses, *n* = 19 Dynasore synapses; ANOVA *p* < 0.00005, Tukey’s *post hoc*). We also examined the effects of monomeric α-synuclein on each stage of clathrin coat formation, which we had not done in our prior study (Busch et al., 2014). Surprisingly, and in contrast to α-synuclein dimers and Dynasore, monomeric α-synuclein impaired a later stage of clathrin-mediated endocytosis during the uncoating step, as shown by a selective increase in stage 4, free CCVs (Figure 6E; *Stage 1*-Control: 0.1 ± 0.1 CCPs; Monomeric α-Syn: 0.2 ± 0.2 CCPs; *Stage 2*-Control: 0.3 ± 0.1 CCPs; Monomeric α-Syn: 0.8 ± 0.3 CCPs; *Stage 3*-Control: 1.4 ± 0.3 CCPs; Monomeric α-Syn: 1.0 ± 0.4 CCPs; *Stage 4*-Control: 0.6 ± 0.2 CCVs; Monomeric α-Syn: 3.7 ± 1.2 CCVs; *n* = 6–14 synapses per condition; ANOVA, *p* = 1.38 × 10^−7^, Tukey’s *post hoc*). Thus, α-synuclein CC and NC dimers selectively inhibited the vesicle fission step during clathrin-mediated SV endocytosis, while monomeric α-synuclein appeared to inhibit clathrin uncoating.

In order to better understand the fission defects, we further examined the morphologies of the CCPs observed in the presence of α-synuclein dimers. Free CCVs were excluded from these analyses. At stimulated control synapses, the vast majority of CCPs were single buds with short, constricted necks, and only a very small number of complex buds with multiple clathrin coats were observed (Figure 6F). However, in the presence of CC and NC dimers, individual CCPs often exhibited longer necks (Figure 6F, top, asterisks). Furthermore, with the α-synuclein dimers, there were larger numbers of complex buds comprising extended membrane tubules ending in multiple clathrin coats (Figure 6F, bottom). These complex budding structures typically emanated directly from the PM (Figure 6F, bottom, arrowheads). A clear example of this can be seen in Figure 6G, where a long, narrow tubule can be traced through two adjacent sections, showing its connection with the PM and a large number of budding CCPs attached (Figure 6G). The individual CCPs induced by α-synuclein dimers also appeared to be morphologically distinct from those observed in the presence of Dynasore or monomeric α-synuclein, which more closely resembled controls (Figure 6F, top). Complex buds and tubules were rarely if ever seen with Dynasore or monomeric α-synuclein treatment under these conditions (Figure 6F, bottom). Quantitatively, there were significantly more single and complex buds at synapses treated with CC and NC dimers, compared to the other experimental conditions (Figures 6H,I; *Single buds—*Control: 2.2 ± 0.2 CCPs; CC dimer: 4.8 ± 0.5 CCPs; NC dimer: 4.8 ± 0.5 CCPs; Dynasore: 3.4 ± 0.4 CCPs; Monomeric α-Syn; 2.1 ± 0.3 CCPs; *p* = 1.46 × 10^−10^; Tukey’s *post hoc*; *Complex buds—*Control: 0.1 ± 0.1 CCPs; CC dimer: 0.8 ± 0.3 CCPs; NC dimer: 1.0 ± 0.4 CCPs; Dynasore: 0.3 ± 0.2 CCPs; Monomeric α-Syn: 0 ± 0 CCPs; ANOVA *p* < 0.001; Tukey’s *post hoc*). When we further examined the necks of individual CCPs, we found no difference in the neck width at synapses treated with α-synuclein dimers, Dynasore, or monomeric α-synuclein, compared to controls (Figure 6J; Control: 31.5 ± 1.1 nm, *n* = 39 CCPs, 28 synapses; α-Syn CC: 28.9 ± 1.2 nm, *n* = 34 CCPs, 10 synapses; α-Syn NC: 35.4 ± 1.3 nm, *n* = 25 CCPs, 12 synapses; Dynasore: 33.3 ± 1.8 nm, *n* = 26 CCPs, 10 synapses; Monomeric α-Syn: 32.0 ± 1.6 nm, *n* = 16 CCPs, 6 synapses; ANOVA *p* = 0.01, Tukey’s *post hoc*). However, both CC and NC dimers significantly increased the neck length on CCPs, compared to all other conditions (Figure 6K; Control: 32.1 ± 1.5 nm, *n* = 39 CCPs, 28 synapses; α-Syn CC: 52.0 ± 2.7 nm, *n* = 34 CCPs, 10 synapses; α-Syn NC: 58.7 ± 3.6 nm, *n* = 25 CCPs, 12 synapses; Dynasore: 28.6 ± 1.8 nm, *n* = 26 CCPs, 10 synapses; Monomeric α-Syn: 27.3 ± 1.6 nm, *n* = 16 CCPs, 6 synapses; ANOVA *p* = 0; Tukey’s *post hoc*). The greater numbers of elaborated complex buds connected by membrane tubules, as well as CCPs with longer extended necks, provides further indication that α-synuclein dimers inhibit vesicle fission during clathrin-mediated SV recycling.

### Monomeric and Dimeric α-Synuclein Induce Distinct Effects on Clathrin-Mediated Endocytosis

**Figure 7:**
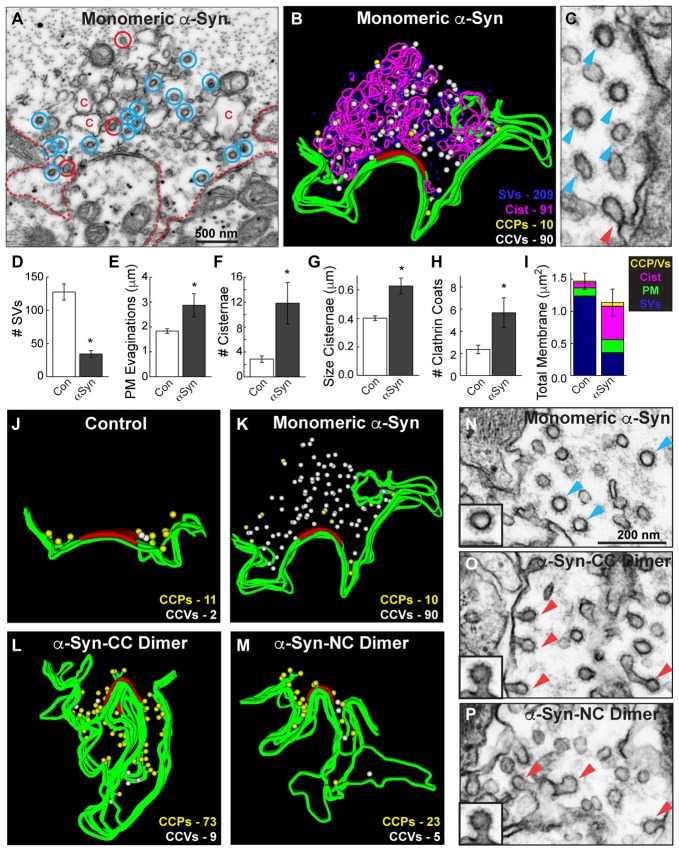
Monomeric and dimeric α-synuclein produce distinct effects on clathrin-mediated SV recycling. **(A)** Electron micrograph showing a stimulated synapse treated acutely with excess monomeric α-synuclein, which causes a reduction in SVs and an expansion of the PM (dotted line). Numerous endocytic intermediates are also observed, including cisternae (C), CCPs (red circles) and free CCVs (blue circles). **(B)** 3D reconstruction of the same synapse. Note the abundance of CCVs (white spheres). **(C)** Inset showing a cluster of CCVs (blue arrowheads) at a synapse treated with monomeric α-synuclein, and only one CCP (red arrowheads). **(D–I)** Quantification of the vesicle recycling defects produced by monomeric α-synuclein. Loss of SVs compensated by a buildup of PM and endocytic intermediates indicates a defect in SV endocytosis. Bars represent mean ± SEM from *n* = 6–14 synapses. Asterisks indicate statistical significance by Student’s *t*-test (*p* < 0.05). **(J–M)** 3D reconstructions comparing the distribution of CCPs and CCVs at synapses treated with monomeric or dimeric α-synuclein. While monomeric α-synuclein induced a buildup of free CCVs (white spheres) throughout the synaptic area, CCPs (yellow spheres) at the PM predominated after treatment with dimeric α-synuclein. **(N–P)** Insets showing the different effects on clathrin-mediated endocytosis. Monomeric α-synuclein impairs clathrin uncoating, as indicated by the buildup of free CCVs (blue arrowheads; inset), and dimeric α-synuclein impairs an earlier stage of endocytosis during the fission step, as indicated by the buildup of CCPs (red arrowheads; insets). Scale bar in **(N)** applies to **(O,P)**.

The analyses above suggested that monomeric and dimeric α-synuclein produced different effects on clathrin-mediated SV recycling, such that the dimeric α-synuclein inhibited an earlier step during vesicle fission while monomeric α-synuclein inhibited a later step during clathrin uncoating. To corroborate this, we further examined the endocytic phenotype produced by monomeric α-synuclein and directly compared it against that produced by dimers. Notably, in the presence of monomeric α-synuclein, free CCVs were abundant (Figure 7A, blue circles), whereas the budding CCPs were sparse (Figure 7A, red circles). This can be seen clearly in Figure 7C where a single CCP (red arrowhead) is surrounded by a cluster of CCVs (blue arrowheads). Clusters of CCVs are highly unusual and are typically due to clathrin uncoating defects, for example after perturbations of the clathrin uncoating ATPase, Hsc70 (Morgan et al., 2001). As reported in our previous study (Busch et al., 2014), monomeric α-synuclein inhibited SV recycling, as indicated by a loss of SVs, which was compensated by an expansion of PM and increased numbers of endocytic intermediates, including cisternae and total clathrin coats (Figures 7D–I; *SVs*: Control: 127.6 ± 12.1 SVs; α-Syn: 34.3 ± 5.0 SVs, Student’s *t*-test *p* = 0.00012; *PM*: Control: 1.8 ± 0.1 μm; α-Syn: 2.9 ± 0.5 μm, Student’s *t-test p* = 0.005; *# Cist*: Control: 2.9 ± 0.5 cisternae; α-Syn: 11.8 ± 3.3 cisternae, Student’s *t-test p* = 0.00082; *Cist Size*: Control: 0.40 ± 0.02 μm; α-Syn: 0.63 ± 0.06 μm, Student’s *t*-test *p* = 0.006; *Clathrin coats*: Control: 2.4 ± 0.4 coats; α-Syn: 5.7 ± 1.4 coats, Student’s *t*-test *p* = 0.005; *Total membrane*: Control: 1.47 ± 0.13 μm^2^, α-Syn: 1.14 ± 0.21 μm^2^, Student’s *t*-test *p* = 0.18; *n* = 14 Control synapses, *n* = 6 α-Syn synapses). While stimulated control, dimer- and Dynasore-treated synapses normally exhibited ~1 CCV per section (Figures 6B–D, Stage 4), in contrast, after treatment with monomeric α-synuclein, the free CCVs were most prevalent (Figure 6E, Stage 4; Figures 7A,C).

A direct comparison of the clathrin coat defects produced by monomeric and dimeric α-synuclein further demonstrated the differences in their phenotypes. We generated 3D reconstructions of synapses where the distributions of CCPs and CCVs are shown in the absence of the other synaptic organelles. These reconstructions clearly revealed that, in comparison to control synapses, monomeric α-synuclein caused an atypical build up of free CCVs that were dispersed throughout the synaptic area (Figures 7J,K, white spheres). Conversely, CC and NC dimer caused a buildup of CCPs at the PM (Figures 7L,M, yellow spheres), and very few CCVs were observed. High magnification electron micrographs clearly revealed the differences in the clathrin-coated structures. Monomeric α-synuclein induced atypical clusters of free CCVs that were separated from the PM (Figure 7N, blue arrowheads). α-Synuclein dimers induced CCPs with constricted necks that were still connected to the PM (Figures 7O,P, red arrowheads). These data provide evidence that monomeric and dimeric α-synuclein affect clathrin-mediated SV recycling at different stages during vesicle endocytosis.

## Discussion

This is the first demonstration that acutely introducing excess levels of α-synuclein dimers to synapses impairs vesicle endocytosis (Figures 4–7). As such, under these conditions, we would consider the dimers to be in the class of “small toxic oligomers”. Two variants of α-synuclein dimers (CC and NC dimers) produced very similar effects at synapses, resulting in a loss of SVs and increased PM area, as well as increased numbers of atypical membrane cisternae and CCPs. Similarly, acute introduction of monomeric α-synuclein has been shown to impair SV endocytosis at both lamprey and mammalian synapses (Figure 7; Busch et al., 2014; Xu et al., 2016; Eguchi et al., 2017). At lamprey synapses, both monomeric and dimeric α-synuclein lead to a rather robust inhibition of SV endocytosis, resulting in a 60%–80% loss of the SV pool (Figures 4–7; Busch et al., 2014). Notably, this phenotype is markedly more severe than is observed after acute perturbations of other regulators of clathrin-mediated endocytosis, such as actin or Hsc70 (Morgan et al., 2004, 2013b; Bourne et al., 2006). Overexpression of α-synuclein in mammalian neurons appears to impair SV trafficking by preferentially affecting vesicle recycling and/or replenishment (Nemani et al., 2010; Scott et al., 2010), though direct effects on exocytosis should continue to be explored (Bendor et al., 2013; Lautenschläger et al., 2017; Logan et al., 2017). We have not yet tested whether acute introduction of excess α-synuclein to lamprey RS synapses directly affects exocytosis, though this is of interest for future studies. Nevertheless, given the measurable effects on clathrin-mediated SV recycling, the current study contributes to a growing body of evidence indicating that increasing levels of α-synuclein at synapses impairs the process of SV recycling.

α-Synuclein dimers inhibited vesicle fission during clathrin-mediated SV endocytosis. This was demonstrated by a selective increase in the numbers of CCPs with constricted necks emanating from the PM, either as individual buds or at the ends of branched membrane tubules (Figure 6). *A priori*, one would surmise that the vesicle fission defect is due to an impairment of dynamin GTPase function. However, under the same experimental conditions Dynasore, a drug that inhibits dynamin GTPase activity, produced measurably different effects from the α-synuclein dimers. First, the build up of CCPs was slightly greater with α-synuclein dimers, compared to Dynasore treatment (Figure 6). Second, the α-synuclein dimers increased the neck length on CCPs, whereas Dynasore did not. Finally, unlike Dynasore, the α-synuclein dimers increased the numbers of complex buds emanating from PM tubules. Thus, α-synuclein dimers additionally appeared to induce membrane tubulation as part of the fission defect, whereas this does not seem to be the case with Dynasore. *In vitro* studies have reported membrane tubulation activity for α-, β- and γ-synuclein (Westphal and Chandra, 2013), and our data would suggest that this is due at least in part by formation of stable dimers on the liposome membranes. Consistent with our findings, in hippocampal neurons and epithelial cells, Dynasore increased the number of late stage CCPs with constricted necks and normal appearance, but conspicuously did not induce long membrane tubules with clathrin coats at their ends (Macia et al., 2006; Newton et al., 2006). Taken together, our data suggest that dimeric α-synuclein produces a vesicle fission defect that is unique and is not simply caused by an inhibition of dynamin GTPase activity. Further studies are needed to determine whether α-synuclein dimers have any other direct or indirect impacts on dynamin function.

Another intriguing finding from this study is that α-synuclein dimers and monomers caused different effects on clathrin-mediated SV endocytosis. We previously reported that acute introduction of monomeric human α-synuclein, or PD-linked point mutant A53T, caused a two to three fold increase in the total numbers of CCPs and CCVs combined at lamprey synapses (Busch et al., 2014). In this study, we further assessed the effects on each morphologically distinct stage of CCP and vesicle formation (Figure 6). Compared to the α-synuclein dimers, which inhibited vesicle fission, monomeric α-synuclein preferentially impaired a later stage of clathrin-mediated SV endocytosis during clathrin uncoating, as shown by an increase in the numbers of free CCVs (Figures 6, 7). In a parallel study, we have determined that the clathrin uncoating defect produced by monomeric α-synuclein is caused by a lack of Hsc70 recruitment to stimulated lamprey synapses; when exogenous Hsc70 is added back to synapses the vesicle trafficking defects are nearly eliminated (Banks et al., 2017). Although we do not yet know the precise molecular mechanism by which α-synuclein dimers impair vesicle fission, the differences between the clathrin coat phenotypes produced by monomeric and dimeric α-synuclein are suggestive of distinct molecular targets.

Metastable α-synuclein multimers have been observed at mammalian synapses under physiological conditions, and they attenuate vesicle release and vesicle recycling (Wang et al., 2014). Immobile microaggregates and SV bound α-synuclein, at least some of which must be multimeric, have also been demonstrated at synapses in a PD mouse model (Spinelli et al., 2014). Thus it is of increasing importance to understand the effects of each molecular species of α-synuclein, their distribution at synapses, and their functions if we are to understand the normal and pathophysiological roles of α-synuclein. Though it is clear that the recombinant dimers are stable and do not form higher molecular weight oligomers *in vitro* according to NMR spectra (Pivato et al., 2012), we do not yet know whether the α-synuclein dimers further multimerize once introduced to the synapse *in vivo*, nor to what extent they partition onto synaptic membranes or remain mobile and cytosolic. However, the fact that the α-synuclein dimers produced a phenotype that was distinct from monomeric α-synuclein, and the fact that one dimer variant (NC) is a single polypeptide, combined with the similarities between CC and NC dimer phenotypes, suggests that the recombinant dimers used in our study do not dissociate into monomers within the synaptic environment. We acknowledge that, unlike our stable α-synuclein dimers, α-synuclein monomers and multimers *in vivo* are likely in a dynamic equilibrium within neurons, undergoing reversible association and disassociation with each other and with membranes. Nonetheless, the strategy of utilizing stable α-synuclein dimers allowed us to further establish effects of α-synuclein at synapses and to begin detecting phenotypic differences with monomeric α-synuclein. This same strategy will therefore be useful for determining the effects of other molecular species of α-synuclein, including tetramers that have recently been described as the native form of the protein (Bartels et al., 2011; Dettmer et al., 2013, 2015a), though this interpretation remains somewhat controversial (Burré et al., 2013). Doing so will allow us to determine the cellular effects of each molecular species of α-synuclein and to probe the underlying molecular mechanisms, providing novel insights into the normal functions of α-synuclein and its pathophysiological effects in disease states. Though PD and other neurodegenerative diseases result from chronic processes, these types of acute perturbation studies will continue to be useful at revealing the direct impacts of excess α-synuclein in the absence of other compensatory effects, thereby elucidating the cellular and molecular machinery to be targeted for possible therapeutic value.

## Author Contributions

All authors (ATM, LGS, IT, LB, JRM) made substantial contributions to the conception and design of the study. ATM, LGS, JRM: data acquisition, data analysis. ATM, LGS, IT, LB, JRM: data interpretation. In addition, LB and IT generated and characterized the recombinant synuclein dimers, which were critical reagents that were essential for the study. All authors (ATM, LGS, IT, LB, JRM) were involved in drafting this manuscript, have provided final approval of this manuscript for submission, and agree to be accountable for all aspects of the work.

## Conflict of Interest Statement

The authors declare that the research was conducted in the absence of any commercial or financial relationships that could be construed as a potential conflict of interest.
